# Collective Phenomena and Non-Finite State Computation in a Human Social System

**DOI:** 10.1371/journal.pone.0075818

**Published:** 2013-10-09

**Authors:** Simon DeDeo

**Affiliations:** 1 Santa Fe Institute, Santa Fe, New Mexico, United States of America; 2 School of Informatics and Computing, Indiana University, Bloomington, Indiana, United States of America; Indiana University, United States of America

## Abstract

We investigate the computational structure of a paradigmatic example of distributed social interaction: that of the open-source Wikipedia community. We examine the statistical properties of its cooperative behavior, and perform model selection to determine whether this aspect of the system can be described by a finite-state process, or whether reference to an effectively unbounded resource allows for a more parsimonious description. We find strong evidence, in a majority of the most-edited pages, in favor of a collective-state model, where the probability of a “revert” action declines as the square root of the number of non-revert actions seen since the last revert. We provide evidence that the emergence of this social counter is driven by collective interaction effects, rather than properties of individual users.

## Introduction

Social systems–particularly human social systems–process information. From the price-setting functions of free-market economies [1], [2] to resource management in traditional communities [3], and from deliberations in large-scale democracies [4], [5] to the formation of opinions and spread of reputational information in organizations [6] and social groups [7], [8], it has been recognized that such groups can perform functions analogous to (and often better than) engineered systems. Such functional roles are found in groups in addition to their contingent historical aspects and, when described mathematically, may be compared across cultures and times.

The computational phenomena implicit in social systems are only now, with the advent of large, high-resolution data-sets, coming under systematic, empirical study at large scales. While such studies are well advanced in the case of both human [9], [10] and non-human [11], [12] communication, these methods have not been widely applied in the study of collective social behavior.

We study a particular phenomenon, that of cooperation in the online, open source Wikipedia community, with the goal of distinguishing between different classes of computational sophistication. We focus on the distinction between finite and non-finite models, where the latter have access to an effectively unbounded resource, such as a counter, stack or queue [13].

A feature common to all such analyses is that a finite amount of data *by itself* can never distinguish between two classes whose distinctions are defined in terms of bounded *vs.* unbounded resources. This is sometimes understood in terms of the competence-performance distinction; see Refs. [9] and [14]. Our argument for the emergence of non-finite computational properties thus relies on model selection, and the statistical inference of asymptotic properties of a finite-state system. As part of this argument we prove a result that we refer to as the *probabilistic pumping lemma*: for any finite-state process, and any string 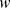, of sufficient length, produced by the process, the probability that a word of length 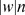 is found to be 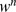 decays exponentially as 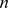 becomes large.

The outline of our paper is as follows. We state, and prove, the lemma described above, in the first section, and Appendix S1 in File S1. We establish the main empirical result of this work in the second section, where we examine the symbolic dynamics of article editing in Wikipedia. In considering the top ten most-edited articles in the encyclopædia, we find strong evidence in a majority of cases for a violation of the probabilistic pumping lemma, and thus computation over and above that of the finite-state.

We then discuss the possible origins of this effectively resource-unbounded system in the third section. We conclude with the implications of this finding for the complexity of social systems, and compare our findings with recent work and explore the analogy between formal grammars and social behavior.

### The Probabilistic Pumping Lemma

In order to distinguish between finite and non-finite models, we focus on the statistics of repeated behavioral patterns, or “words”. In this section, we show explicitly that probabilistic finite-state process have an exponential cutoff in the asymptotic distribution of repeated words.

Our discussion here relies on the properties of 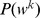 or, in words, “the probability of the word 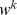
”, or, more explicitly, “the probability that a randomly drawn string of length 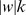 will be 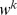.” Measurement of 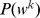 from data is non-trivial, and detailed discussion of this appears in Appendix S3 in the File S1.

Our proof establishes the existence of an exponential cutoff by showing that the limiting ratio of 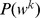 (the probability of observing the word 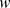 repeated 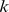 times in a sample of length 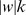), and 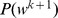, as 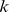 becomes large, approaches a constant strictly between zero and one. We will be able to determine that limiting constant in terms of the properties of the underlying system.

#### Statement of lemma

For any probabilistic finite-state process, any initial distribution over internal states, and any word 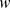, where (1) for all 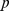 there exists a 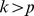 such that 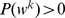 and (2) the system does not deterministically repeat a single word, there exists a positive real number ε such that.
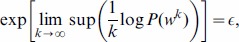
(1)as 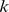 becomes large, with 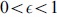, 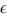 *strictly* greater than zero and *strictly* less than one. The limiting value, 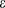, is the spectral radius of 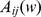, the natural extension of the symbol transition matrix to multi-letter words.

The complete proof is given in Appendix S1 in File S1. Tests of the numerical convergence of this relation are presented in Appendix S2 in File S1, where we study how small machines (number of states of order ten) converge to the bound of Eq. 1 for a uniform prior over spectral radius.

Informally, the lemma says that 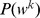 is bounded above by an exponential cutoff of the form 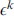, 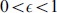. For most processes, the relevant scale for the limit to obtain is 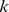 of order 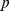, the number of states in the underlying process.

Given this, and under the mild assumption that the system has passed through its transient states to one of its aperiodic final classes, the asymptotic probability 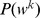 takes the form of a sum of exponentials,
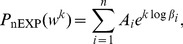
(2)where here 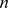 is the number of classes, and 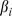 are all strictly between zero and one. Eq. 2, which we refer to as the nEXP model, forms the basis of our model comparisons, and the evidence for non-finite-state computation, presented in the next section.

Note that, for the special case of a purely deterministic (non-probabilistic) machine, where each state has only one transition, either (1) 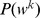 will be zero for all 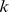 greater than some fixed value or (2) the output string will just be repetitions of 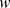; either violates the conditions of the lemma. Deterministic machines can be recognized by looking for exact repetitions; the more general case that violates Eq. 2, aperiodicity, can be recognized by non-monotonic behavior.

Note also that the absence of a violation of the probabilistic pumping lemma is not evidence against non-finite-state computation. Even in the case of infinite data, it is easy to construct non-finite-state processes that show exponential decay in all repeated strings; an example can be constructed for a stochastic context-free language that generates strings of matched, but arbitrarily nested, parentheses: “…( )((( )) ( ))…”.

### The Case of Wikipedia

We now consider a real-world example of collective behavior in a human social system. We are interested in the underlying computational structure of the process, and in particular, the question of whether the system might have access to an unbounded resource. To that end, we compare an infinite-resource model to the general finite-state case using model selection.

### 1. Model Selection

A finite-state model, given a sufficient number of states, can reproduce the statistics of an arbitrary process. In statistical study, one must therefore ask when the data justify a simpler (if non-finite) model with fewer parameters. This is known as *model selection*.

Model selection provides a principled and self-consistent way to select between different descriptions of a process, and to determine (among other things) when adding additional parameters to a model is justified. Without model selection, it would be impossible to establish the existence of a power-law (as opposed to a sum of exponentials), a sine function (as opposed to a finite number of terms in its Taylor series expansion), or a linear trend (as opposed to a truncation of its Fourier decomposition).

Model selection is often done informally, based on the intuitive appeal of one model over another. Here, we attempt a more rigorous approach based on Bayesian methods. The Bayes factor, which provides a self-consistent method for model selection, is now in wide use in the biological [15], [16] and physical sciences [17]–[21]. It is of particular use when the question concerns selection between competing hypotheses, rather than (as happens in the frequentist paradigm) the rejection of a null hypothesis [22].

For model selection, there are two relevant quantities. The first is 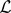, the log-likelihood of the posterior, or the log of the probability of the data given the best choices of parameters for the model in question,
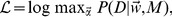
(3)where 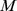 is a particular model, 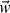 is the vector of parameters associated with 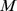, and 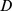 is the data. Models of sufficient generality can, with sufficiently many parameters, make 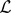 arbitrarily large for a given data-set.

The second quantity, 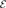, is the Bayesian evidence for the model, or, the log-likelihood of the data averaged over all possible parameter values,
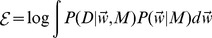
(4)


It is the Bayesian evidence 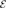 that allows us, in a consistent fashion, to select between models; the reader is referred to Ref. [23]. Meanwhile, the log-likelihood 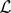 is useful as a diagnostic to see which features of the data are relevant.

The Bayesian evidence requires use of a prior, 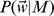; careful specification of the prior is necessary to avoid unfairly penalizing one model over another. In both models we consider, parameters may specify (1) an overall normalization, (2) relative amplitudes of different components, or (3) timescales of decay. We place uniform priors on normalization and decay timescales (within reasonable bounds), and model the priors for relative amplitudes as uniform on the simplex.

To compute 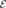, we use a standard approximation (Ref. [23]; see Appendix S4 in the Supporting Information File). This quantity can be directly interpreted as the log-probability in favor of a model, given the data; thus 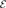, the difference between 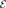 for two models, corresponds to the log probability in favor of one model versus the other.

### 2. Article Timeseries Data

We consider the “edit history” of encyclopædia articles, taken individually. These histories amount to a time-series of editor behaviors: the time-stamped changes to the page made by individuals (either anonymous, or pseudonymous).

Coarse-graining of these histories is necessary: the number of possible edits that editors can make is essentially unbounded and any edit may change, add, or delete arbitrary amounts of text from the article. A well-known distinction, however, exists between edits that alter the text in a novel fashion and those that “roll back” the text to a previous state. The latter kind of edit, called a “revert” is used when an editor disagrees with an edit made by someone else and, instead of altering the text further, undoes the work of his or her opponent; as we describe below, revert edits are strongly correlated in time with conflict, and are themselves considered anti-social actions in the context of normal editing.

We thus coarse-grain the history of edits made on an article into two classes, R (“revert”) and C (“cooperate”: any non-revert edit). An example of this process is shown in Table 1, while the details of our processing of the raw data are given in Appendix S3 in the Supporting Information File.

**Table 1:** A day of edits on the George_W._Bush page, starting at midnight UTC, 21 March 2006.

time (UTC)	user	SHA1 (partial)	code
02∶08	Sarah	4abc4aef1ea5	**C**
05∶02	Alexh25	1e3a2a4656d8	**C**
05∶04	Mhking	4abc4aef1ea5	**R**
11∶39	Trezatium	3b03700b0d9c	**C**
12∶15	Brazilfantoo	94a5c05ba10e	**C**
12∶31	Brandon39	3b03700b0d9c	**R**
23∶28	Titoxd	109986b8f390	**C**
23∶31	Titoxd	334a315944ce	**C**
23∶38	Titoxd	739c15e5bc6a	**C**
23∶40	Titoxd	3063a0289680	**C**
23∶42	Titoxd	7aafc8f3f762	**C**

As can be seen by comparing SHA1 hashes of the page content, user Mhking reverted an edit by user Alexh25 to the previous version by user Sarah. Later in the day, user Brandon39 reverted user Brazilfantoo. In between, one can see “cooperative” stretches involving both single and multiple users. This sequence of events is coarse-grained into the substring “CCRCCRCCCCC.” The full string of (in this case) 45,220 action symbols forms the basis of the finite-state analysis. As with all data used in this study, this sequence is publicly available, in this case at http://en.wikipedia.org/w/index.php?title=George_W._Bush&offset=200603218&action=history [last accessed 15 August 2013].

A feature of Wikipedia relevant to this binary classification of edits into revert and non-revert is the presence of so-called “vandalism”–improper and non-constructive modifications or blanking of the page. Since they usually do not take the form of reversion, these would be classed as C. More detailed descriptions (“prosocial non-revert ” *vs.* “antisocial non-revert”) and similarly for the revert case, where pro-social reverts repair vandalism, are certainly possible, and, from the point of view of a detailed understanding, desirable.

At a coarse-grained level, however, revert edits are a natural class to consider in a study of online conflict [24]–[26]. As noted by Ref. [27], who studied reversion as a measure of conflict across multiple Wikipedia-like systems, reversions capture implicit cases of task conflict, which are strongly associated with the broader phenomenon of relationship conflict [28]. Within the Wikipedia community itself, reverts are considered signs of conflict [29], as can be seen in widely accepted social norms such as the “three revert rule” that encourage editors to find ways of resolving conflicts, rather than undoing each other's edits [30].

We focus on the most-edited pages, since these provide the greatest amount of data and allow for the most detailed distinctions to be made between pages. While there are large numbers of much less-edited pages, we believe that more sophisticated statistical methods would be required to aggregate this data in such as way as to make statistical study at this level possible.

### 3. Two Models

We consider two conceptually distinct models.

The first model is finite; in particular, we consider a finite-state model class of sufficient generality–the probabilistic finite-state machine–that it contains every other model on the finite side of the finite-infinite divide of the computational hierarchy. We consider the probability of seeing an unbroken run of 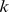 cooperative events, 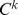, given that we have just seen a revert, 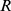. By the probabilistic pumping lemma, it has the asymptotic form.
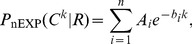
(5)where 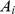 and 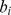 are free parameters that specify the amplitude and decay rate (timescale) of the 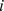
th independent component, and 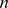 specifies the number of components.

The second model we refer to as the *collective state* model. In this model, the probability of an additional cooperative event, C, has a functional dependence on the number of cooperative events seen preceding. It is easiest to formulate as the probability of an unbroken run of length 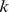,
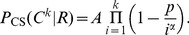
(6)


In words, the collective state model allows for increasing “returns to scale”: as the number of cooperative events increases, the probability of a non-cooperative event declines as a power-law with index 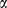.

Underlying mechanisms have a natural description in the collective state model. In particular, the probability of seeing a *non*-cooperative action, conditional on already having seen 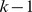 cooperative actions just previously,
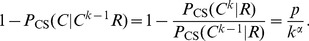
(7)scales as a power-law with index 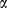. For example, if 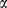 is close to unity, then, the collective state model says that the probability of a non-cooperative action declines linearly with the amount of cooperation seen previously. The particular values of 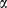 found in the data thus have a direct interpretation in terms of potential underlying mechanisms.

As is clear from Eq. 6, the collective state model violates the probabilistic pumping lemma. It is thus, formally, non-finite. Intuitively, the state space of this model is an effectively unbounded counter that increments with each cooperative event, and resets with each revert.

## Results

**Figure 1:**
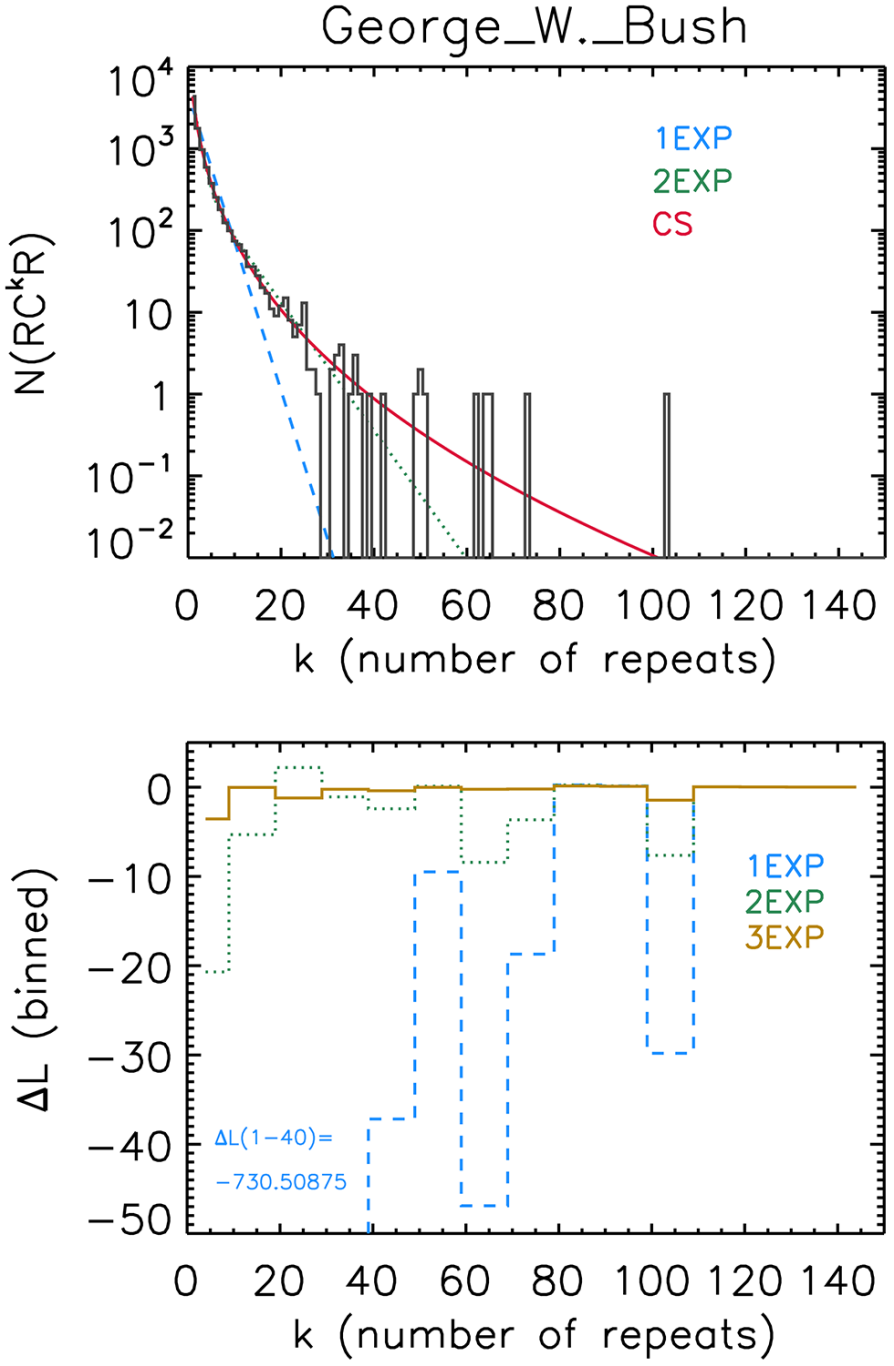
Top. Distribution of consecutive C (“cooperative”) events in the edit history of the most-edited article on the English-language Wikipedia, George_W._Bush. Solid histogram: actual data. Red/solid line: maximum-likelihood fit for the three-parameter collective state (CS) model of Eq. 6, preferred over the sum of exponential model (nEXP) of Eq. 2. The blue/dashed and green/dotted lines show the one and two component finite-state approximations to the Collective State model. The finite state model approximates the collective state model in this data at four components (eight parameters), at which point it is strongly disfavored as non-parsimonious by Bayesian model selection. **Bottom**. Contributions to 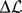 (log-likelihood relative to collective state) for the one, two, and three component fits (blue/dashed, green/dotted and yellow/solid, respectively).

Fig. 1 shows the distribution of consecutive C edits for the most edited article in the Wikipedia “main space” (*i.e.*, that set of pages supposed to constitute the encyclopædic content): that referring to George W. Bush, the 43rd President of the United States. We refer the reader to Appendix S3 in File S1, where we show that counts of the number of strings of the form 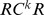, written 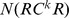, is the preferred data to estimate from.

Even at a glance it is clear that a single exponential–which would appear as a straight line on a log-linear plot–is insufficient to describe the decay of 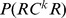 as a function of 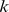. However, visual inspection alone is insufficient to determine whether to prefer a sum of exponentials (Eq. 2) to an explicitly non-finite-state process, and we present in Table 2 the log evidence ratio, 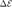, in favor of the collective state model. This table shows that strong evidence against the nEXP model, and in favor of the collective state model, can be found in a majority of cases of the top-ten most-edited articles on the encyclopædia.

**Table 2:** log-Evidence (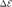) ratios, for the collective state versus the finite-state case, for the ten most-edited pages on Wikipedia.

sig.	page name	historylength	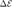	collectivestate index
			CS *vs.* nEXP	*α*
<10^−8^	George_W._Bush	45,220	18.5	0.576±0.005
<10^−6^	Islam	18,054	14.9	0.592±0.007
<10^−5^	United_States	31,919	12.3	0.545±0.006
	Global_warming	19,541	12.1	0.602±0.008
<10^−4^	Wikipedia	31,927	11.3	0.638±0.006
	Michael_Jackson	26,977	10.4	0.572±0.007
<10^−3^	2006_Lebanon_War	19,656	9.1	0.49±0.01
	Deaths_in_2009	20,902	7.7	0.42±0.01
>10^4^	Deaths_in_2007	18,215	−11.5	–
>10^7^	Deaths_in_2008	19,072	−17.5	–

In cases where the collective state model is strongly favored (large, positive 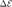), we show the best-fit value of the 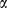 parameter (see Eq. 6). Eight pages show strong (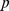-value 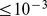) evidence for the collective state (CS) model of Eq. 6 over and above that for the sum of exponentials (nEXP). The strongest evidence in *favor* of finite-state computation is found for two of the three “death list” pages, which collate otherwise unrelated information from other parts of the encyclopedia. Appendix S4 in the File S1 gives details on the use and computation of 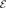 for model selection.

Table 2 also presents the collective state index 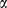. We find that, in cases where the data favor the collective state model, this index is between 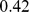 and 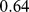; the average value in the top-ten is 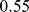. Eq. 7 allows us to interpret this index in terms of the rate at which non-cooperative actions become less likely.

Our results thus show that the probability of a cooperative run being terminated by a revert action *declines roughly as the square-root of the number of cooperative events* seen in that run. Whatever the underlying nature of the unbounded resources governing the time-series, they must at least be able to maintain a counter, incremented with each C symbol seen, and reset with each R.

### Origins of Memory in the Collective State

In this section, we conduct additional analyses to determine properties of the system that might give clues to the nature of the underlying process.

The results of the previous section provide strong statistical evidence (odds ratios greater than 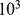) for preferring a non-finite model to an explicit enumeration of timescales. The cases in Table 2 for which this is *not* the case are themselves of interest. These articles are of a very different nature: “death lists,” collections of single sentences listing the dates of deaths of noteworthy individuals.

That these cases are better described by the sum-of-exponentials model suggests that the article content is relevant to the emergence of non-finite-state computation. This can be either because the user bases that particular content-types attract make it easier for the resultant system to produce non-finite-state behavior. Or, conversely, it could be that the article content itself leads to non-finite-state editing patterns.

It could be the case that the cumulative effects associated with the functional form of Eq. 6 come from non-interacting users who independently and separately come into contact with an article. The interactions between individuals, on this picture, are unimportant; the content of the page (or a single user's own memory) serves as an effectively unbounded resource that allows violation of the exponential cutoffs required by the finite-state case.

For example, upon interacting with the page cooperatively, the user might alter it in such a way as to make the probability of a second cooperative edit (by the same user) more likely, and so on. Such a process could potentially lead to behaviors of the same nature as those accounted for by the CS model, without having anything to do with any interpersonal or group-level interaction.

**Figure 2:**
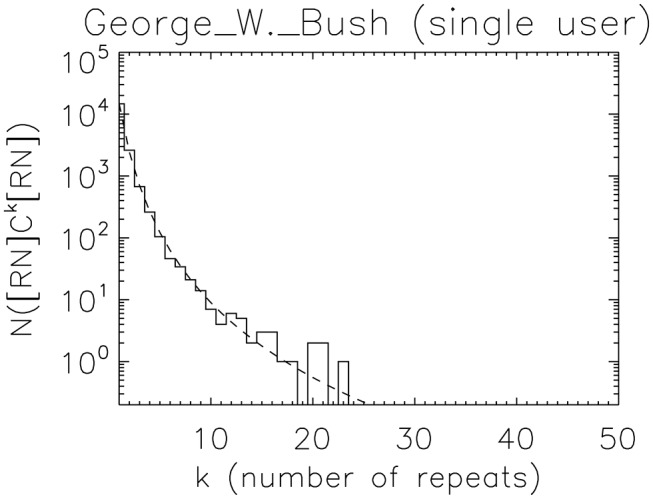
Solid line: distribution of consecutive *single-user* C (“cooperative”) events in George_W._Bush. The contrast to the multi-user case is clear, showing that long periods of cooperative editing can not be accounted for by unbroken single-user patters. The distribution is well-modeled by the collective state model, Eq. 8, with distinct functional form and parameter values from the fit for the multi-user case. The fit is preferred to the finite-state nEXP model at 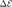 (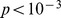).

Fig. 2 examines this question in detail for the George_W._Bush case. We now augment the time-series with an additional symbol, N, representing a change of user (for example, for the data shown in Table 1, the new series would be CNCNRNCNCNRNCCCCC), and count strings of consecutive Cs bracketed either by R or N; in other words, a change of user is considered to interrupt the run of Cs. We find the CS model preferred at the 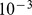 level over nEXP; interestingly, the particular functional form of the CS model is the simpler, limiting case.
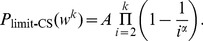
(8)


This non-exponential form is not necessarily evidence for non-finite computation in any particular individual; the distribution found for the collection could be understood as the superposition of finite-state machines drawn from a distribution representing the spread of the properties of individuals.

The distinct functional form of the distribution at the individual level suggests that some aspect of interpersonal interaction plays a role in the non-finite nature of the full process. Whether this is driven by how groups are more able to take advantage of the effectively unbounded resource of the page itself (a “large scratchpad” model), or because some system memory is encoded in the interactions between the users themselves (an “interaction combinatorics” model) is an open question.

An obvious visual difference between Figs. 1 and 2 is the elimination of the long tail; it so turns out that long cooperative runs are multi-user events. While it is not the case that long cooperative events necessarily imply the collective state (CS) over the nEXP model (they can be found as well in the “death list” pages, where they are fit by a single long timescale exponential component), it is certainly true that the exponential decays implied by the probabilistic pumping lemma require increasingly unlikely fine-tunings of amplitude and decay constants to fit long periods of cooperative behavior.

In the particular case of the George W. Bush page associated with the analysis in this section, the preference for a collective state model in both the individual and the collective case suggests we postulate not one, but at least two distinct counters: one that increments with each C, and is reset with each R, and a second one that increments with each C, and is reset with each R or N.

## Conclusions

This work has examined cooperative behavior in a large-scale social system. We have examined competing models for the processes we observe, and found strong statistical evidence in favor of a *collective state* model. Despite the non-finite nature of the underlying process, the collective state model is more parsimonious than competing finite-state models that approximate it. At the most coarse-grained level of analysis, this model requires at least one “counter” that alters the structure of the system over time.

The results comparing collective and individual editing properties further suggest that distinct mechanisms for the violation of the finite-state case are associated with, on the one hand, the cognitive properties of individuals taken separately, and on the other, the fundamentally social phenomenon of Wikipedia as a whole. Distinct counters appear to be running in parallel.

The underlying mechanisms responsible for the emergence of these counters is an open question. They may be fundamentally connected to reputation or memory effects [31]–[33]; alternatively, full accounts may require attention to the emergence of social norms [34], [35]. Our results here suggest ways to modify and extend “tit-for-tat” models of behavior in social systems [36] by means of counters that track more fine-grained aspects of system state. In addition to these social context effects, the task itself may play a crucial role: the content of the page itself may itself shift the behavior of editors.

This paper has relied on the use of formal languages. First applied to the case of human language [9], they have now been extended to describe human social interaction (see, *e.g.*, Ref. [37] on “shaking hands”), animal communication [12], [38], animal behavior [39] and pattern recognition more generally (Ref. [10] and references therein). This joins the empirical study of cognitive phenomena to a long tradition in the theory of complexity [40].

When the state of a group is taken to be the sum of the states of the individuals that compose it, coarse-grainings of the system state will in general lead to effective theories [41] whose basic units are not descriptions of the state of any one individual. We have previously given such accounts in the case of an animal system [42], [43], where a single formalism is used to attribute computational (“strategic”) states to both individual animals and emergent groups. Ref. [44] provides an explicit analogy between the formal language hierarchy and the decompositions of Ref. [42].

Our work in this paper extends these accounts to human social systems, considered not as ensembles of individual (formal) language users but as a free-standing and unreduced process. Over and above its role in the discussion about cooperative phenomena in social systems, our main result presents a challenge to theory: what formalisms are most natural for the description of non-finite-state processes in the biological and social world?

Our results demonstrate that empirical study itself can play a role in determining the relative importance of different ways a system can transcend the finite-state aspects of a system: large scratchpads *vs*. interaction combinatorics. While formal language theory presents us with a number of “post-finite” languages, such as the context-free grammars and pushdown automata [13], it seems likely that these will have to be extended or modified to provide tractable models for empirical investigation.

## Supporting Information

File S1 **Contains four appendices.** Appendix S1: Proof of the Probabilistic Pumping Lemma; Appendix S2: Numerical Tests of Convergence Properties; Appendix S3: Details on Coarse-Graining and Analysis of Wikipedia Behavior; Appendix S4: Details on Model Selection.
(PDF)
