# Correction: Collective Phenomena and Non-Finite State Computation in a Human Social System

**DOI:** 10.1371/journal.pone.0101511

**Published:** 2014-06-23

**Authors:** The PLOS ONE Staff

## Notice of Republication

This article was republished on May 23, 2014, to replace an incorrectly published version in which there were symbol errors throughout the PDF and XML of the article. Please download this article again to view the correct version. The originally published, uncorrected article and the republished, corrected article are provided here for reference.

## Supporting Information

File S1 Originally published, uncorrected article.
(PDF)

File S2 Republished corrected article.
(PDF)
