# Supplementary material for: Collective Phenomena and Non-Finite State Computation in a Human Social System
Source: PLoS One. 2013 Oct 9;8(10):e75818. doi: 10.1371/journal.pone.0075818 (PMC3794014; doi:10.1371/journal.pone.0075818)
Supplement: File S1 — Contains four appendices. Appendix S1: Proof of the Probabilistic Pumping Lemma; Appendix S2: Numerical Tests of Convergence Properties; Appendix S3: Details on Coarse-Graining and Analysis of Wikipedia Behavior; Appendix S4: Details on Model Selection. (PDF) [file pone.0075818.s001.pdf]

Supporting Information for Collective Phenomena and Non-Finite State Computation in a Human Social System

Simon DeDeo[1,2,*]
1 Santa Fe Institute, Santa Fe, NM 87501, USA
2 School of Informatics and Computing, Indiana University, Bloomington, IN 47405, USA
$*$ E-mail: simon@santafe.edu

Appendix S1: Proof of the Probabilistic Pumping Lemma

Statement of Lemma. For any probabilistic finite-state process, any initial distribution over initial states, and any word w, if there exists a p such that for all $k > p$, $P(w^k) > 0$ [possibility condition], and the process is not simply a deterministic repetition of a single word w, there exists a positive real number ϵ, $0 < \epsilon < 1$, such that $\exp[\lim_{k\rightarrow\infty} \sup{(1/k)} \log P(w^k)] = \epsilon$ as k becomes large.

Proof. We will assume the Mealy machine formalism (observed symbols are emitted upon transitions between internal states [1]). Let A be the transition matrix for the process; an element $A_{ij}(\sigma)$ gives the conditional probability of a transition to state j, emitting symbol σ, given that one was previously in state i. If the process is reducible, we will assume that sufficient time has passed for the process to reach irreducible subspace of this matrix, and we confine our attention to that subspace.

We may extend the definition of $A(\sigma)$ to words, as

$$A_{ij}(w) = \sum_{a_1,\ldots,a_{|w|}} A(w_0)_{ia_1} A(w_1)_{a_1 a_2} \cdots A(w_{|w|})_{a_{|w|}j},$$

where w_i is the ith symbol in word w. We have, further,

$$0 < A_{ij}(w) \leq A_{ij}^{|w|}, \tag{1}$$

or, in words, the probability to go from state i to state j and emit the word w is less than or equal to that of simply going from i to j in the same number of steps.

By the Perron-Frobenius theorem, the inequality of Eq. 1 implies that all eigenvalues, β_i, of $A_{ij}(w)$ are within the unit circle ($|\beta_i| \leq 1$ for all i) with equality obtaining only in the case that $A_{ij}(w)$ is identical to $A_{ij}^{|w|}$. We neglect this latter, trivial case, which only obtains when w is shift-invariant and the all observation runs are given by repeated instances of w. Conversely, the possibility condition amounts to the condition that the matrix $A_{ij}(w)$ is not nilpotent, and there exists a non-zero eigenvalue.

If the system (or our knowledge of it) is distributed over its internal states according to probability vector π_i, we can write the probability of observing a repeated string w as a trace,

$$P(w^k) = \sum_{i,j=1}^{n} \pi_i A_{ij}^k(w). \tag{2}$$

While we have assumed for simplicity that A_{ij} is irreducible, this will not usually be the case for $A_{ij}(w)$. This latter matrix will in general contain both essential and inessential "self-communicating" classes[1] along with a set of nuisance indices that connect to no other class (*i.e.*, i for which $A_{ij}(w)$ is equal to zero for all j) [3].

The structure of $A_{ij}(w)$ may be visualized as a directed acyclic graph. Inessential classes may have non-zero out-degree, while essential classes, and nuisance indices, are the terminal nodes. Self-loops are permitted, and exist for both inessential and essential classes; these will be crucial to our argument below.

[1] An index i leads to an index j (written $i \rightarrow j$) iff there exists a k such that $A_{ij}^k(w) > 0$. Indices i and j communicate if $i \rightarrow j$ and $j \rightarrow i$. Communication is an equivalence relation, so that classes can be built that contain indices that communicate with each other. Essential classes (sometimes called "final" classes [2]) are those which do not lead to any index outside the class; inessential classes are those which may.

Because the initial distribution π may have zero entries, we consider only the part of $A_{ij}(w)$ corresponding to descendants of the non-zero part of π in the associated directed acyclic graph. Transitions among the set of nuisance indices, by definition, can not repeat an index. Thus their structure is not relevant to the asymptotic behavior of $P(w^k)$, and we may focus on the essential and inessential classes.

We are particularly interested in the classes that will dominate the $P(w^k)$ probability as k becomes large. Consider the restriction of $A_{ij}(w)$ to a particular class α: *i.e.*, construct a submatrix from $A_{ij}(w)$ using only $i, j \in \alpha$. Call this restriction $\alpha_{ij}(w)$. Consider, similarly, the restriction of the distribution π to this class.

Assume first that $\alpha_{ij}(w)$ is diagonalizable. Then, the probability of producing k copies of w, while remaining in the class α, is

$$P(w^k|\alpha) = \sum_{i,q=1}^{|\alpha|} \beta_q^k \pi^{(q)} v_i^{(q)}, \tag{3}$$

where β_q is the qth eigenvalue of $\alpha(w)$, and

$$\pi_i = \sum_{q=1}^{|\alpha|} \pi^{(q)} v_i^{(q)}. \tag{4}$$

By construction of the equivalence classes, α is irreducible. Then, by the Perron-Frobenius theorem, the largest eigenvalue of this matrix, β_1, is real, has a strictly positive eigenvector, and $\pi^{(1)}$ is necessarily greater than zero.

If $\alpha_{ij}(w)$ is acyclic then $P(w^k|\alpha)$ can be written

$$P(w^k|\alpha) = A_1 \beta_1^k \left(1 + \sum_{i=2}^{\alpha} A_i \left(\frac{\beta_i}{\beta_1}\right)^k\right), \tag{5}$$

where $A_1 > 0$, β_1 is real, and $|\beta_i| < \beta_1$ for all $i > 1$, and

$$\exp\left[\lim_{k\to\infty}\left(\frac{1}{k}\log P(w^k|\alpha)\right)\right] = \beta_1. \tag{6}$$

If $\alpha_{ij}(w)$ is diagonalizable, but the period, d, is greater than one, we will have additional eigenvectors associated with complex rotations of β_1, $\beta_1 \exp 2\pi i k/d$, $k = \{1 \ldots d-1\}$. These will lead to additional oscillatory terms in the leading order term; these oscillations of will be governed by an overall exponentially-decaying envelope, so that

$$\exp\left[\lim_{k\to\infty}\sup\left(\frac{1}{k}\log P(w^k|\alpha)\right)\right] = \beta_1, \tag{7}$$

regardless of the period of $\alpha_{ij}(w)$.

Finally, consider the case of non-diagonalizable $\alpha_{ij}(w)$. In this case, the matrix can be brought into Jordan normal form, with m blocks, each of size n_i and associated with an eigenvalue β_i. Assume that the matrix is aperiodic. By the Perron-Frobenius theorem, n_1 is equal to one [4]. The kth power of $\alpha_{ij}(w)$ can then be written (see, *e.g.*, Ref. [5]),

$$P(w^k|\alpha) = A_1 \beta_1^k + \sum_{i=2}^{m}\left(\sum_{j=0}^{n_i-1} A_{ij}\binom{k}{j}\beta_i^{k-j}\right), \tag{8}$$

where $A_1 > 0$, β_1 is real, and $|\beta_i| < \beta_1$ for all $i > 1$ as before. When k is greater than the largest block size, we can write

$$P(w^k|\alpha) = A_1 \beta_1^k \left(1 + \sum_{i=2}^{m} f_i(k)\left(\frac{\beta_i}{\beta_1}\right)^k\right), \tag{9}$$

where $f_i(k)$ is a polynomial function of k, of degree $n_i - 1$. Eq. 8 thus obeys Eq. 6; for a non-aperiodic α, an argument identical to the above gives the convergence of Eq. 7.

Having understood the single-class case, we now consider w^k strings generated by multiple classes.

Any particular string w^k may be generated by a set of transitions within and between classes. Because these transitions are governed by the directed acyclic graph structure, there will be a finite number of transitions between states. Thus, as k becomes large, the probability of $P(w^k)$ for a particular set of transitions will be governed by the self-transitions, given by terms of the form Eq. 7.

In particular, $P(w^k)$ is the sum of a finite number of terms; each term in the sum is a product of at most p transitions between classes, and at least $k-p$ terms of the form $P(w^n|\alpha)$, for different α. Explicitly,

$$P(w^k) = \sum_{i \in p(G)} T_i \prod_{j=1}^{N} P(w^{n_{i,j}}|\alpha_j), \tag{10}$$

where i indexes the paths of length k through the graph G representing the underlying $A_{ij}(w)$ structure, T_i is a prefactor governing the probabilities of transitions between classes, N is the number of classes, and the total number of within-class transitions is forced to grow with k,

$$\sum_{j=1}^{N} n_{i,j} \geq k - p \tag{11}$$

for all possible paths i.

For large k, the growth in the number of possible paths (*i.e.*, the growth of the $|p(G)|$) is bounded by the growth in the number of ways to partition the sum in Eq. 11. In particular, for large k, the number of possible paths relevant to $P(w^k)$ can increase only polynomially in k.[2]

Meanwhile, each term in the sum of Eq. 10 is decreasing exponentially, governed by products of the $\beta_{i,1}$, the largest eigenvalues for the classes that have self-transitions for that term. The dominant terms in the sum will be those for which the exponential decline is slowest. By the Perron-Frobenius theorem, the largest eigenvalue of a submatrix associated with a class of $A_{ij}(w)$ is equal to the spectral radius of the matrix as a whole. If $P(w^k)$ is greater than zero for k larger than p, the pigeonhole principle invoked in the ordinary pumping lemma [6] allows us to assume the existence of at least one self-communicating class; this then means that the spectral radius is equal to that of $A_{ij}(w)$ itself.

$$0 < \exp\left[\lim_{k\to\infty} \sup\left(\frac{1}{k}\log P(w^k)\right)\right] = \rho(A_{ij}(w)) < 1, \tag{12}$$

which was to be proved. □

While our paper presents the first explicit application of this form of reasoning to human social systems, we note in passing the use of this kind of reasoning in the study of bird song. Once regarded as strictly finite-state [7], the sound sequences produced by songbirds are now recognized to show features of non-finite-state computation. A recent, compact model of song production in the Bengalese finch (*Lonchura striata domestica*) [8], demonstrates the need for a self-modifying (and thus non-finite-state) Markov process.

An analysis of data on a different species, the Zebra finch (*Taeniopygia guttata*), shows that the probability of an additional repetition, the analog of this paper's $P(C^{k+1})/P(C^k)$, decreases exponentially [9]. This is, of course, the other way to violate the probabilistic pumping lemma (under the assumption of having reached an aperiodic final class)—the exponential of the lim-sup, Eq. 7, goes to zero as opposed to unity. It is just as much evidence against finite-state computation, but found in the anomalous absence, rather than presence, of extreme events.

[2] For any set of transitions between classes, the number of self-transitions is bounded by k, and the number of distinct classes to assign those self-transitions to is bounded by p, the number of states in the machine. The number of ways p terms can sum to k is $\binom{k-1}{p-1}$, which is bounded by the polynomial k^p.

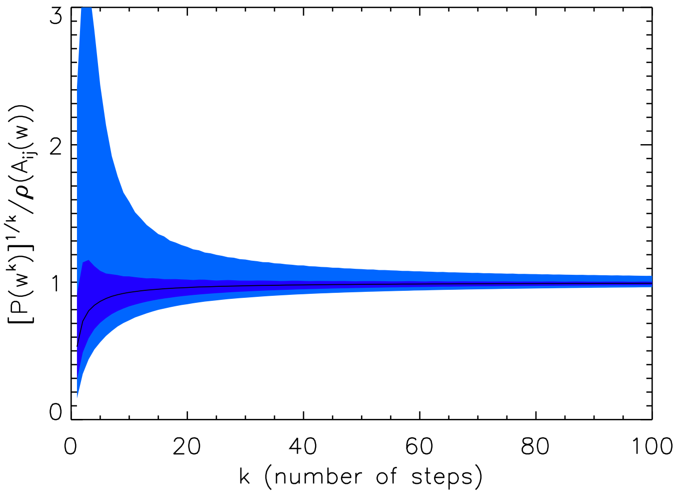

Figure 1. Numerical study of convergence of repeated word frequencies to exponential decay, with cutoff predicted by the spectral radius. Shown here is the measured decay rate to the asymptotic limit predicted by Eq. 12, for irreducible finite-state processes with ten states, two output symbols $\{C, R\}$, w equal to C, and a uniform distribution over values of $\rho(A_{ij}(w))$, the spectral radius and asymptotic decay rate, between $0 < \rho < 1$. Light blue shows 2σ, and dark blue 1σ ranges about the median value. For empirical work, convergence is much faster when considering $[P(w^{q+k})/P(w^q)]^{1/k}$, with q larger than the (assumed) number of states.

Appendix S2: Numerical Tests of Convergence Properties

With a view towards determining how the lemma of the previous section applies to actual finite-state processes, we study a restricted class of machines numerically. We sample from the space of probabilistic unifilar machines with p states over a two-symbol alphabet. Such a system can be represented by a weighted, directed graph, with each node having at least one, and at most two outgoing edges, each of which is associated with one of the two symbols, and whose weights sum to unity.

For small p, the underlying graph-theoretic space can be described completely: for each node, we have a choice of one vs. two outgoing edges; in the case of only one outgoing edge, we must choose between the two symbols. Neglecting the possibility of equivalent machines, we then have the number of such machines, as a function of p, as

$$N(p) = (2p + p^2)^p, \tag{13}$$

which grows rapidly: there are 12 billion such machines with six states, and more than 10^{400} with one hundred states.

We are most interested in how quickly the statistics of an actual machine approaches the limiting value given by Eq. 12. For any particular $A_{ij}(w)$, we can compute the spectral radius and compare that to the ratio $P(w^k)/P(w^{k-1})$ found for distributions over initial conditions that include a self-communicating class as a function of k.

In Fig. 1 we show convergence to the limit by sampling the space of strongly-connected ten-state machines, and considering the frequency of a single repeated symbol. We take a uniform prior over $\rho(A_i(w))$, the spectral radius and limit established by the lemma of the previous section, and show the

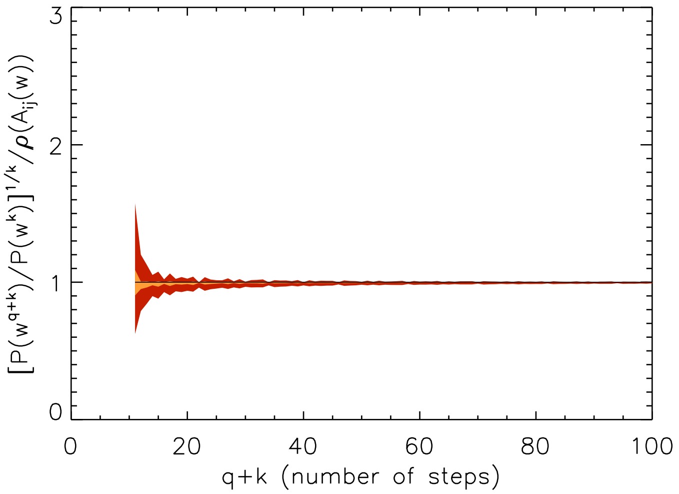

Figure 2. Convergence to exponential cutoff as seen with $\hat{C}(q,k)$ (Eq. 15), for the same system as in Fig. 1. Here we take q equal to ten, the number of states. For the same amount of data, convergence is faster for $\hat{C}$ than C; here convergence for $\hat{C}$ to the asymptotic value (at 1σ confidence), is achieved for k equal to thirty.

convergence ratio, *i.e.*,

$$C(k) = \frac{[P(w^k)]^{1/k}}{\rho(A_{ij}(w))}, \tag{14}$$

to provide a numerical example of the limiting process established in the previous section. For small k, $P(w^k)$ may be dominated by movement through nuisance states and inessential classes, and by contributions from essential classes that have small self-communication probability. Convergence to the spectral radius thus occurs much faster when considering

$$\hat{C}(q,k) = \frac{[P(w^{q+k})/P(w^q)]^{1/k}}{\rho(A_{ij}(w))}, \tag{15}$$

where q is longer than the relevant scales of the transient phenomena (*e.g.*, at least as large as the assumed number of states.) This is shown explicitly in Fig. 2, where we take q to be the number of states in the system.

Appendix S3: Details on Coarse-Graining and Analysis of Wikipedia Behavior

Our coarse-graining of behaviors on any particular page aims at locating where one user reverts (undoes) the contributions of another editor completely. We locate reversion edits in two distinct ways. Firstly, following Ref. [10], we can identify reversion edits by the presence of keywords, such as `rv` and `revert`, in the edit summaries; we do so with the following regular expression: `/([Rr][v]+[\ \n]|[Uu]ndid|[Rr]evert)/`. Secondly, following analyses such as those of Ref. [11], we can look for versions of a page with identical SHA1 checksums; the version with the later timestamp may thus be considered a revert to the earlier page. In general, these two metrics align very well, although not perfectly; in this work, we focus on the

latter method as a more objective one that does not rely on editors self-reporting. We do not include self-reverts, or edits that do not alter any aspect of the page (*i.e.*, that would otherwise look like "reverts to the current version").

The probabilistic pumping lemma works in terms of $P(w^k)$, and our analysis considers the probability of repeated cooperation. However, the measurement of $P(C^k)$ in the data, if done naively, leads to unacceptable results. In particular, estimating $P(C^k)$ for a particular page by counting the number of times the string C^k appears in the time-series, leads to strong bin-to-bin correlations, since an observation of a string C^k necessarily leads to observations of strings of the form $C^{k-1}, C^{k-2}, \ldots, C^{k-\lfloor k/2\rfloor+1}$, and then *two* observations of the form $C^{k-\lfloor k/2\rfloor}$, and so on. This would lead to excessive complications in the likelihood analysis; conversely, if the correlations are neglected, it leads to claims of heavy-tailed distributions that spuriously rule out exponential decay.

Instead, we count prefix- and suffix-free strings that do not have this shift problem—in particular, we consider the quantity $N(RC^kR)$. As long as $N(RC^kR)$ is significantly less than N, counts of RC^kR and RC^mR are independent of each other and we can write

$$P(RC^kR) \approx \frac{N(RC^kR)}{N}.$$

The quantity $P(RC^kR)$ itself can be written as

$$\begin{aligned} P(RC^kR) = P(R)P(C^k|R)P(R|RC^k) &= P(R)P(C^k|R)\left[1 - P(C|RC^k)\right] \\ &= P(R)\left[P(C^k|R) - P(C^{k+1}|R)\right]. \end{aligned} \tag{16}$$

In the case that $P(C^k|R)$ is the sum of exponentials in k, we have

$$N(RC^kR) \propto P(C^k|R) \propto P(C^k), \tag{17}$$

or, in words, that if $P(C^k)$ is a sum of exponentials, so is $N(RC^kR)$. The relationship between these two quantities is not always so simple; in the collective state (CS) case, Eq. 16 implies that the quantity $N(RC^kR)$ has a different functional form from $P(C^k)$. In particular, we have

$$P_{\mathrm{CS}}(RC^kR) = \frac{Ap}{(k+1)^\alpha}\prod_{i=1}^{k}\left(1 - \frac{p}{i^\alpha}\right), \tag{18}$$

which is the functional form we fit and display in Fig. 1 of the Main Article.

Appendix S4: Details on Model Selection

In this section we describe in greater detail our methods for distinguishing between the asymptotic and exponential models.

Computation of the likelihood ratio requires an error model for the distributions of $N(RC^kR)$. Since we lack an explicit model for the errors themselves, as a first approximation, we take measurements of $N(RC^kR)$ to be identically and independently distributed. For $N(RC^kR) \ll N$, N the total number of observations, this is a reasonable assumption. Given this, the Poisson distribution of counts follows, and computation of $\mathcal{L}$, the log-Likelihood, or $\log P(D|\vec{w}, M)$, for any particular model M with parameters $\vec{w}$, can be written as

$$\Delta\mathcal{L} = \sum_{k=1}^{k_{\max}} N(RC^kR)\log\lambda(\vec{w}, k) - \lambda(\vec{w}, k), \tag{19}$$

where we drop model-independent constants. Given sufficiently flat priors, $P(\vec{w}|M)$, around the peak of this function, this is sufficient to estimate many quantities of interest, including the maximum *a posteriori* values of $\vec{w}$ and the error bars on those estimates.

Our main goal, however, is not parameter estimation, but rather *model selection*, where one compares models with different parameter spaces. In our particular case, one class of models (nEXP) can approximate, by superposition of exponentials, the other class (CS). As the number of exponentials in the sum increases, the approximation becomes increasingly good. We would like to know when we are justified in preferring the more parsimonious model.

Two main frameworks for the resolution of this question exist. On the one hand, the Aikiake Information Criterion (AIC) can be used to estimate the expected KL divergence between the predictions of a model and the true process. In the limit of large amounts of data, it prescribes a constant penalty of k, the number of parameters, to the likelihood.

This penalty is sometimes taken as an "Occam penalty," but the correct interpretation is as a guide for prediction out of sample. Prediction out of sample is a conceptually distinct problem, since a complicated approximation to the true model may work very well in a limited range, particularly in the presence of experimental noise. In Monte Carlo testing, AIC tends to prefer complicated approximations, even in cases where the underlying model is more parsimonious [12]; a related formal result is that AIC is "dimensionally inconsistent," meaning that even in the limit of infinite data, use of the AIC will lead to non-zero probability of choosing an (incorrect) approximation [13].

On the other hand, one can compute (or approximate) what is called the Evidence[3], which requires knowledge of both the likelihood, $P(D|\vec{w}, M)$, and the prior expectation of parameter ranges, $P(\vec{w}|M)$,

$$E = P(D|M) = \int P(D|\vec{w}, M)P(\vec{w}|M)\ d^k w, \tag{20}$$

where k is the number of parameters (dimensionality of $\vec{w}$). Formally, the Evidence is proportional to "the probability of the model M, given the data observed," if equal prior probability is given to the models under consideration. As in all model selection cases, absolute values of the Evidence are irrelevant. One considers only ratios and phrases the question, as in Table 1, as to whether (for example) "model A is at least a factor of 10^3 more likely than model B."

In this work, we take the latter approach, operating entirely within the Bayesian framework. This is because our contrasting model classes have small numbers (less than ten) of parameters, all of which have clearly specifiable priors, $P(\vec{w}|M)$. Computation of the full posterior is now common when these circumstances obtain, as is often the case in the exact sciences [14–16].

In order to calculate E, we use the Laplace (or saddle point) approximation; in log-units,

$$\begin{aligned}\mathcal{E} = \log E \approx\ & \mathcal{L}(\vec{w}_{\max}) + \log P(\vec{w}_{\max}|M) \\ & -\frac{1}{2}\log\det A + \frac{1}{2}k\log 2\pi,\end{aligned}$$

where $\mathcal{L}$ is the log-likelihood, $\vec{w}_{\max}$ are the parameters that maximize the likelihood, and A is the Hessian, equal to

$$A_{ij} = -\left.\frac{\partial^2\mathcal{L}}{\partial w_i \partial w_j}\right|_{\vec{w}_{\max}}. \tag{21}$$

We refer the reader to Ref. [17] for details on this approximation.

It remains to specify the priors $P(\vec{w}_{\max}|M)$ for the two models. The nEXP class has $2n$ parameters; the CS class has 3. The parameters are of two kinds.

Both nEXP and CS have parameters corresponding to the one-step decay of the underlying quantity $P(C^k)$. In the case of nEXP, there are n such parameters, b_i, that play this role. In the case of CS, there is only one, p. We take a uniform prior in p (CS) and b_i (nEXP). We allow all p to range independently

[3] A common rough approximation to this function gives the BIC, or "Bayesian Information Criterion," which prescribes a penalty of $n \log |D|$, where $|D|$ is the amount of data.

between zero and 0.995; the high end corresponds to an exponential cutoff of order 200 repeats, much longer than seen in the data.

We then have normalizations of terms (n normalizations for nEXP, one for CS). These are fixed by the value of $P(C^1)$, the overall cooperative fraction.

$$N(C) \approx NP(C). \tag{22}$$

The maximum value of $P(C)$ is unity. This then leads to an overall area factor of

$$\frac{N^n}{n!}, \tag{23}$$

for nEXP, where the factor of $n!$ is because the overall sum of all normalizations is confined to the interior of an N-dimensional simplex. In the case of CS, $P(C^1)$ is equal to $A(1-p)$. We thus have to integrate over the range of p values to find the area associated with the CS normalization prior,

$$N \int_0^{0.995} \frac{1}{1-p}\, dp \approx 5.29832N. \tag{24}$$

Finally, CS has a third parameter, α. For each value of $1-p$, we allow this to range between zero (pure exponential) and $\alpha(p)$, where $\alpha(p)$ is set to give a $1/e$ cutoff at 200 repeats. As an example, $\alpha(0.995)$ is zero; if α were greater than zero, the overall function would have an exponential cutoff longer than 200 repeats. Given these, the area factor for nEXP is 0.995^n, and for CS is it

$$\int_0^{0.995} \alpha(p)\, dp \approx 1.28841. \tag{25}$$

Putting together all these area factors, we can then pre-compute $-\log A$, equal to $\log P(\vec{w}_{\max}|M)$, a constant independent of $\vec{w}$. For the `George_W._Bush` article, for example, we have $-\log A$ equal to -12.6 for the CS case, and -10.3 (1EXP), -18.7 (2EXP), -27.4 (3EXP). Note that prior areas are not directly comparable between different models; "change of units" (*e.g.*, working in terms of $P(RC^kR)$ vs. $N(RC^kR)$) will scale A. This scaling, however, is directly compensated for by the Hessian determinant term.

Together with the max log-likelihood, the determinant of the Hessian, and the $+k\log 2\pi$, these are sufficient to compute the (Gaussian approximation to) the relative log-Evidence for the two model classes $\Delta\mathcal{E}$, reported in Table 1 (Table 2 in the Main Paper). In general, the highest evidence member of the nEXP class is either 3EXP or 4EXP. Table 1 gives the results for the top thirty most-edited pages.

sig.	page name	history length	$\Delta\mathcal{E}$ CS *vs.* nEXP	collective state index α
$< 10^{-8}$	George_W._Bush	45,220	18.5	0.576 ± 0.005
$< 10^{-6}$	World_War_I	14,808	15.9	0.521 ± 0.009
	Islam	18,054	14.9	0.592 ± 0.007
$< 10^{-5}$	Iraq_War	15,143	12.8	0.60 ± 0.01
	Scientology	14,584	12.2	0.595 ± 0.009
	United_States	31,919	12.3	0.545 ± 0.006
	Global_warming	19,541	12.1	0.602 ± 0.008
$< 10^{-4}$	Australia	13,815	11.4	0.679 ± 0.009
	Wikipedia	31,927	11.3	0.638 ± 0.006
	September_11_attacks	17,253	11.3	0.530 ± 0.008
	Gaza_War	14,764	11.3	0.45 ± 0.01
	Israel	16,319	11.1	0.523 ± 0.008
	Super_Smash_Bros._Br	15,343	11.1	0.451 ± 0.008
	Turkey	14,384	11.1	0.501 ± 0.009
	List_of_Omnitrix_ali	16,263	10.6	0.450 ± 0.008
	Michael_Jackson	26,977	10.4	0.572 ± 0.007
	Canada	17,670	9.4	0.632 ± 0.008
	Blink-182	14,419	9.4	0.461 ± 0.009
$< 10^{-3}$	2006_Lebanon_War	19,656	9.1	0.486 ± 0.009
	Blackout_(Britney_Sp	15,714	7.9	0.348 ± 0.009
	Deaths_in_2009	20,902	7.7	0.416 ± 0.009
$< 10^{-2}$	Heroes_(TV_series)	14,060	6.6	0.353 ± 0.009
	Xbox_360	16,598	6.4	0.498 ± 0.009
	Lost_(TV_series)	14,714	5.1	0.387 ± 0.008
	Paul_McCartney	16,649	4.7	0.72 ± 0.01
(no det.)	Eminem	17,417	4.3	—
	Pink_Floyd	15,730	2.9	—
	Deaths_in_2006	14,072	0.8	—
$> 10^{4}$	Deaths_in_2007	18,215	-11.5	—
$> 10^{7}$	Deaths_in_2008	19,072	-17.5	—

Table 1. log-Evidence ratios for the thirty most-edited pages on Wikipedia. Computed from data last accessed 15 July 2013.

References

1. Hartmanis J, Stearns RE (1966) Algebraic structure theory of sequential machines. Prentice-Hall international series in applied mathematics. Prentice-Hall.

2. Berman A, Plemmons RJ (1987) Nonnegative Matrices in the Mathematical Sciences. Classics in Applied Mathematics. Society for Industrial and Applied Mathematics. Ch. 2.3.

3. Seneta E (2006) Non-negative Matrices and Markov Chains. Springer Series in Statistics. Springer. Ch. 1.2.

4. Meyer C (2000) Matrix Analysis and Applied Linear Algebra. SIAM Press. Ch. 8.

5. Andrieux D (2011) Spectral Signature of Nonequilibrium Conditions. arXiv preprint arXiv:11032243 .

6. Pippenger N (1997) Theories of Computability. Cambridge University Press.

7. Berwick RC, Okanoya K, Beckers GJL, Bolhuis JJ (2011) Songs to syntax: the linguistics of birdsong. Trends in Cognitive Sciences 15: 113–121.

8. Jin DZ, Kozhevnikov AA (2011) A compact statistical model of the song syntax in Bengalese finch. PLoS computational biology 7: e1001108.

9. Liberman M (2011). Finch linguistics. In *Language Log*, `http://languagelog.ldc.upenn.edu/nll/?p=3261`. Informal analysis of data supplied by Ofer Tchernickovski and Dina Lipkind. Last accessed 15 August 2013.

10. Kittur A, Kraut RE (2010) Beyond Wikipedia: Coordination and Conflict in Online Production Groups. In: Proceedings of the 2010 ACM conference on Computer supported Cooperative Work. Savannah, GA,: ACM Press, p. 215.

11. Yasseri T, Sumi R, Rung A, Kornai A, Kertész J (2012) Dynamics of conflicts in Wikipedia. PLoS ONE 7: e38869.

12. Kass RE, Raftery AE (1995) Bayes Factors. Journal of the American Statistical Association 90: 773–795.

13. Liddle AR (2004) How many cosmological parameters? Monthly Notices of the Royal Astronomical Society 351: L49–L53.

14. Mortonson MJ, Peiris HV, Easther R (2011) Bayesian analysis of inflation: Parameter estimation for single field models. Physical Review D 83: 43505.

15. Easther R, Peiris HV (2012) Bayesian analysis of inflation. II. Model selection and constraints on reheating. Physical Review D 85: 103533.

16. Noreña J, Wagner C, Verde L, Peiris HV, Easther R (2012) Bayesian analysis of inflation. III. Slow roll reconstruction using model selection. Physical Review D 86: 23505.

17. MacKay DJC (2003) Information Theory, Inference and Learning Algorithms. Cambridge University Press. Ch. 28.
